# Validation of SMOS Soil Moisture Products over the Maqu and Twente Regions

**DOI:** 10.3390/s120809965

**Published:** 2012-07-25

**Authors:** Laura Dente, Zhongbo Su, Jun Wen

**Affiliations:** 1 Faculty of Geo-Information Science and Earth Observation (ITC), University of Twente, Hengelosestraat 99, 7514AE Enschede, The Netherlands; E-Mail: b_su@itc.nl; 2 Key Laboratory of Land Surface Process and Climate Change in Cold and Arid Regions, Cold and Arid Regions Environmental and Engineering Research Institute (CAREERI), Chinese Academy of Sciences, Lanzhou 730000, China; E-Mail: jwen@lzb.ac.cn

**Keywords:** soil moisture, SMOS, validation, Tibetan Plateau, Maqu, Twente

## Abstract

The validation of Soil Moisture and Ocean Salinity (SMOS) soil moisture products is a crucial step in the investigation of their inaccuracies and limitations, before planning further refinements of the retrieval algorithm. Therefore, this study intended to contribute to the validation of the SMOS soil moisture products, by comparing them with the data collected *in situ* in the Maqu (China) and Twente (The Netherlands) regions in 2010. The seasonal behavior of the SMOS soil moisture products is generally in agreement with the *in situ* measurements for both regions. However, the validation analysis resulted in determination coefficients of 0.55 and 0.51 over the Maqu and Twente region, respectively, for the ascending pass data, and of 0.24 and 0.41, respectively, for the descending pass data. Moreover, a systematic dry bias of the SMOS soil moisture was found of approximately 0.13 m^3^/m^3^ for the Maqu region and 0.17 m^3^/m^3^ for the Twente region for ascending pass data. Several factors might have affected the retrieval accuracy, such as the presence of Radio Frequency Interference (RFI), the use of inaccurate land cover information and the presence of frozen soils not correctly detected in winter. Improving the RFI filtering method and the quality of the retrieval algorithm inputs, such as land surface temperature and land cover, would certainly improve the accuracy of the retrieved soil moisture.

## 1. Introduction

The Soil Moisture and Ocean Salinity (SMOS) satellite carrying the Microwave Imaging Radiometer with Aperture Synthesis (MIRAS), passive microwave 2-D interferometric radiometer, was launched in November 2009. The main aim of this European Space Agency (ESA) mission is to provide global maps of soil moisture and ocean salinity [1,2]. MIRAS is able to provide measurements of brightness temperature at the L-band (1.4 GHz) for a range of viewing angles from 0° to 55° and with a spatial resolution of 35 to 50 km [3].

The main innovations of the MIRAS radiometer, compared to other radiometers currently in orbit, are the operating band and the new antenna system. The L-band was selected for several reasons: higher sensitivity to soil moisture and ocean salinity, lower attenuation of atmosphere and vegetation and larger penetration depth in the soil layer, than encountered at higher frequencies in the microwave range. To date, the use of the L-band was limited by requiring a very large antenna, but this was overcome in MIRAS by employing a synthetic aperture antenna. Another reason of operating in L-band was that this is a protected radio band, therefore the measurements should be free of interference. Unfortunately, since the beginning of the mission, the SMOS brightness temperature measurements revealed the presence of man-made sources contaminating the natural emission from several areas of the Earth [4]. Studies on the detection and filtering of Radio Frequency Interference (RFI) in the SMOS data are ongoing and RFI is currently considered the most important source of error in the SMOS products.

The SMOS soil moisture retrieval algorithm is based on the inversion by minimization approach. This approach consists of minimizing the difference between the actual SMOS measurements and the brightness temperature estimated by a direct model of the surface by means of a cost function, knowing the land cover and soil texture [5].

As both the SMOS sensor technology and the retrieval algorithm are new, there is currently uncertainty about the quality and reliability of the generated soil moisture products. For this reason, the validation of the SMOS soil moisture products is of crucial importance, not only to investigate the achievement of the mission objectives, but also in view of possible applications of these data and of future satellite missions for soil moisture retrieval in the L-band such as the Soil Moisture Active and Passive (SMAP) mission. Several measurement networks and campaigns were set up in order to assess the accuracy of the SMOS soil moisture products, and the performance of the SMOS retrieval over these sites is currently under investigation. Based on the results obtained by these studies, the SMOS retrieval algorithm will be refined. Therefore, it is expected that the quality and accuracy of SMOS L2 soil moisture products will be improved.

The main objective of the present study is to contribute to the accuracy assessment of the currently available SMOS soil moisture products (reprocessed data, version 5.05 of the Level 1 processor and version 5.01 of the soil moisture Level 2 processor). The SMOS retrieval performances were investigated in two regions: the Maqu region on the Tibetan Plateau in China and the Twente region in The Netherlands. Due to its high elevation, the Tibetan Plateau has a strong influence on the climatic system in Asia. As soil moisture is one of the key variables in hydrological and climatic studies, there is a need for accurate soil moisture information collected at a large scale by satellite sensors over the Plateau. The SMOS data are suitable for these studies, therefore their validation over the Tibetan Plateau is very important. The Maqu region is characterised by a mixture of flat and mountainous topography and by a homogenous land cover. The Twente region was chosen as validation site, because complementary to the Maqu region, as it has a flat topography and heterogeneous land cover. The two regions also differ in climate. The differences between Maqu and Twente region allow for different aspects of the validation of satellite-derived soil moisture to be analyzed. Extensive networks were set up in both regions to continuously monitor the soil moisture and soil temperature at 5 cm depth, as well as in deeper layers, to provide the information necessary for the validation of SMOS products and of other satellite-derived soil moisture products. Both networks consist of 20 stations and span an area larger than one SMOS resolution cell. This is an important feature that partially overcomes the problem of the large gap in scale between *in situ* soil moisture measurements and satellite-derived soil moisture estimates.

## 2. Materials

### 2.1. Maqu Dataset

**Figure 1.**
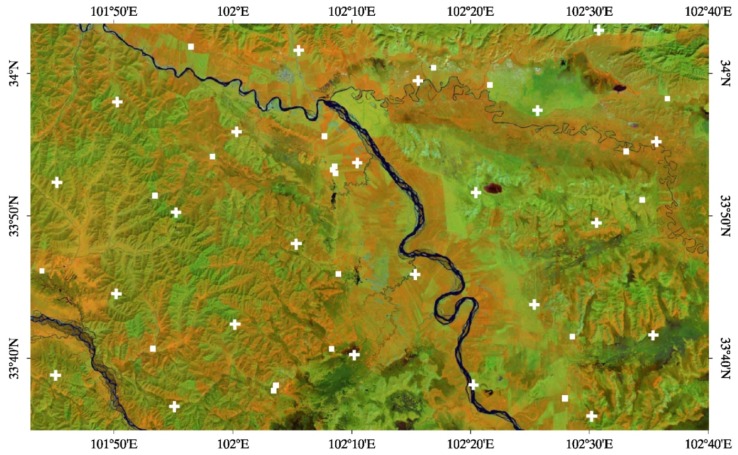
Color composite (R: band 4—G: band 5—B: band 1) of a Landsat 5TM image over the Maqu region (short vegetation in green, light brown and orange; forested areas in shades of reds and dark browns; urbanized areas in cyan; water bodies in dark blue). The location of the monitored sites is indicated by a white rectangle and the centre of the SMOS nodes by a white cross.

The Maqu soil moisture monitoring network was set up in July 2008 on the north-eastern fringe of the Tibetan Plateau (33°30′–34°15′N, 101°38′–102°45′E), located in the southern part of Maqu county, on the border between Gansu and Sichuan provinces in China. A detailed description of the network is reported in Dente *et al.*, [6] and in Su *et al.*, [7]. The network is located at the first major bend in the Yellow River, where the landscape is characterised by the large river valley and surrounding hills with an elevation ranging from 3,200 m to 4,200 m a.s.l. The Maqu region is shown in Figure 1 by a Landsat 5TM image collected in September 2007, with the locations of the monitored sites highlighted as white rectangles. The land cover consists of uniform short grassland with silt loam soils and the wetlands cover a large part of the valley. The climate is characterised by dry and cold winters (November–March) and by a rainy and relatively warmer monsoon season (April–October). Soil moisture and soil temperature are continuously measured by means of EC-TM ECH_2_O probes (Decagon Devices, Inc., USA) at a depth of 5 cm (and in deeper layers) at 20 sites every 15 min. The monitoring sites are distributed over an area of approximately 40 km × 80 km and are characterised by a variety of altitudes and slopes and differing soil characteristics. The network set up ensures that the soil moisture spatial variability of the Maqu region is well monitored, and the spatial average of the measurements collected at each site can be considered an accurate indicator of the soil moisture dynamics at the network scale, as shown in [6].

A detailed land cover map is available for the Maqu region, the Multi-source Integrated Chinese Land Cover (MICLCover) map provided by the Cold and Arid Regions Environmental and Engineering Research Institute of the Chinese Academy of Sciences (CAREERI/CAS). The MICLCover map was obtained by integrating several classification maps over China including a vegetation map, a land use map for the year of 2000, a swamp-wetland map, a glacier map and a Moderate-Resolution Imaging Spectroradiometer land cover map for 2001 (MODIS2001) using a decision-fuse method based on the Dempster-Shafer evidence theory [8]. The accuracy of the MICLCover map was assessed at 71% all over China, which was shown to be higher than the MODIS2001 map accuracy.

### 2.2. Twente Dataset

**Figure 2.**
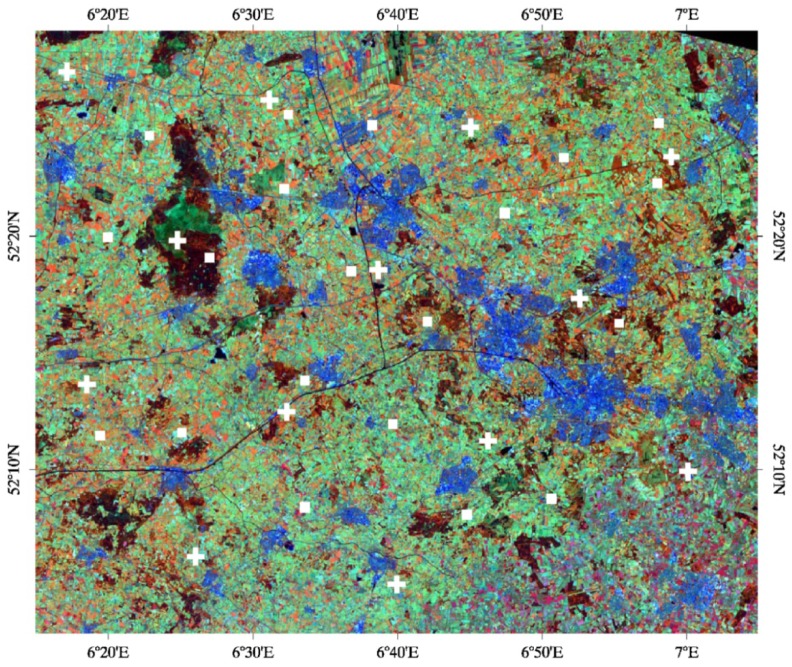
Same as Figure 1 but for the Twente region.

The Twente soil moisture monitoring network is located in the eastern part of the Overijssel province in The Netherlands, mainly covering the region called Twente, but also part of the Salland region and the Gelderland province (52°05′–52°27′N, 6°05′–7°00′E). The region is flat, with an elevation ranging between 3 m to 50 m a.s.l. The most extensively occurring land cover is grassland for pasture which is harvested and fertilized several times a year. However, the land use of this region also includes a mosaic of agricultural fields, forest patches and several urban areas. A Landsat 5TM image collected in June 2010 of the Twente region is shown in Figure 2, with the locations of the monitored sites highlighted as white rectangles. The main crop is corn, which is planted in April and harvested in September. In this region, the soil texture of the surface layer is mainly sand and loamy sand. Soil moisture and soil temperature have been continuously monitored since July 2009 at 20 sites, spread across an area of approximately 50 km × 40 km at a depth of 5 cm every 15 min. The sites were selected in order to monitor the area extensively for a variety of soil types and land covers. However, one of the main factors affecting the spatial variability of soil moisture in this area, besides land cover, is the ground water table. Due to groundwater management occurring in this region, the groundwater table may rise to 25 cm below the soil surface as well as drop to below 160 cm. EC-TM ECH_2_O probes (Decagon Devices, Inc., USA) are used to measure the soil moisture and temperature at 5 cm depth, as well as in deeper layers. A detailed description of the network and the dataset is reported in Dente *et al.*, [9].

### 2.3. SMOS L2 Soil Moisture Products

SMOS Level 2 (L2) soil moisture products (reprocessed data, version 5.05 of the Level 1 processor and version 5.01 of the soil moisture Level 2 processor) from January 15 to December 15, 2010 were used in this study. The products are geolocated on an equal-area grid called Icosahedral Snyder Equal Area (ISEA), where the inter-cell distance is uniformly 15 km. The equator crossing time of ascending and descending passes is at 6 a.m. and 6 p.m. (local solar time), respectively, and the complete globe is covered every 3 days.

An iterative approach is followed in order to retrieve soil moisture and vegetation optical thickness. This approach is based on the principle of finding the optimal set of soil moisture and vegetation parameters that minimizes the difference between modeled and measured brightness temperature [5] for a variety of incidence angles, for a given surface cover and soil texture. The obtained soil moisture is an estimate for the surface layer of the soil, a few centimeter deep [10]. The retrieval algorithm is described in detail in the Algorithm Theoretical Basis Document [11]. The forward model used in the SMOS Level 2 processor is the L-band microwave emission of the biosphere (L-MEB) model [12]. L-MEB is based on a simplified zero-order radiative transfer equation [13], where the rough soil surface contribution to the brightness temperature is modeled following the approach by Wang and Choudhury [14] and the vegetation contribution is modeled following the *τ* − *ω* approach, *i.e.*, the optical depth *τ* and the single scattering albedo *ω* are used to parameterize the vegetation attenuation and the scattering effects within the canopy layer. The soil effective temperature is computed as a function of soil temperature at the soil surface and at increasing depth, as described in Wigneron *et al.*, [15]. The soil dielectric mixing model by Dobson *et al.*, [16] is used to relate the soil permittivity to soil parameters such as soil moisture.

The retrieval is carried out at each node of the ISEA grid and the parameterization of L-MEB depends on the dominant land cover at the node and its surrounding area. Information on the land cover class along with soil and vegetation properties are provided to the SMOS L2 processor by the ECOCLIMAP land surface global database [17] at a resolution of 1 km.

The land cover map in the ECOCLIMAP database is obtained by integrating various data sources, including two global land cover datasets: the International Geosphere-Biosphere Programme Data and Information System (IGBP-DIS) [18,19] and the University of Maryland database (UMd) [20]. The location of the surfaces permanently covered by ice and wetlands is provided by the IGBP-DIS database, whereas all the other cover types are derived from the UMd global land cover map.

In the L2 processor working area of 123 km × 123 km surrounding each node (the distance of 123 km corresponds to twice the largest extent of the 3 dB footprint occurring in SMOS soil moisture observations), different land cover types may be present, contributing to the surface microwave emission. Therefore, the weight of each contribution is determined taking into account the cover fraction and the SMOS radiometric sensitivity in the antenna pattern. In this way, the dominant cover type is assessed and if it consists of a surface type where soil moisture estimation is meaningful (for example low vegetation or forest), retrieval is carried out. For the non-dominant cover type of the node, the contribution to the overall emissivity of the node is taken into account by the L-MEB model, but no retrieval is performed. Any given node is covered by several views, each having a different spatial extent. Therefore, the application of the weight function may give different results for each view.

Auxiliary data, such as soil temperature at the surface and at depth, initial soil moisture values and information about snow cover, are provided by the European Centre for Medium-Range Weather Forecasts (ECMWF) model. When the ECMWF surface soil temperature drops below a given threshold, the soil is considered frozen and soil moisture data are not retrieved. When, according to the ECMWF model, dry snow is present, then the snow is considered transparent and retrieval is carried out as if there was no snow.

The L2 soil moisture products contain not only the retrieved parameters (soil moisture and optical thickness), but also ancillary information obtained during the retrieval, such as surface temperature, roughness parameters and dielectric constant, as well as several confidence, processing and science flags. The confidence and processing flags refer to the quality and characteristics of the retrieval, retrieval options and conditions adopted during the processing and information on L1c data used for the retrieval. The flags useful for the analysis carried out in this study include those indicating: if the retrieval values were outside the expected range; if there was a poor fit between modeled and observed brightness temperature; if the retrieval error was above a certain threshold. The science flags refer to features of the surface that might affect retrieval, for example presence of snow, frozen soils, wetlands and forest. Information about the suspected presence of RFI can be obtained from two bands of SMOS L2 products, N_RFI_X and N_RFI_Y, reporting, for any given node, the number of brightness temperature measurements in X and Y polarization, respectively, disregarded in the retrieval process because of suspected RFI. Combining this information with the total number of brightness temperature measurements available for the given node (M_AVA0) provides the percentage of brightness temperature measurements where RFI is suspected.

## 3. Validation Method

There are 13 ISEA grid nodes included in the area of the Twente monitoring network and 23 nodes in the area of the Maqu monitoring network and soil moisture is nominally retrieved for each of them. The locations of the SMOS grid nodes covering the regions are highlighted in Figures 1 and 2. In order to validate the SMOS soil moisture over these two networks, the spatial average of the values estimated at all nodes included in the network areas was computed. The average was taken when SMOS data were available for at least three nodes. A time series of SMOS average soil moisture was obtained in both regions between January 15 and December 15, 2010. The data analysis was carried out separately for products retrieved from SMOS ascending data to those retrieved from descending data, because these are characterized by a different acquisition time and eventually by different RFI effects, as they each have a different swath coverage.

Firstly, the general seasonal behavior of the time series of the SMOS data spatial average was compared to the corresponding *in situ* spatial average of the measurements at 5 cm depth. Then, several factors were investigated.

If SMOS retrieval is only carried out at a few nodes in the network areas, the spatial variability of the soil moisture might be not well monitored. Therefore, it was checked if the SMOS data covered at least half the area of interest and it was investigated if SMOS soil moisture inaccuracies were related to a partial coverage of the SMOS data.

The reason why SMOS data were not retrieved over a part or the entire area of interest was sought in the flags that are raised when no product is generated, such as the flag for frozen surface, the flag for retrieved values outside the expected range and the flag for high retrieval error. The percentage of nodes with one of these raised flags was computed when missing retrieval interested a part of the area of interest and the entire area, by dividing the number of nodes with a raised flag by the total number of nodes without data. The consistency of the frost flag with the *in situ* measurements of soil temperature was investigated, as well. The information concerning the suspected presence of RFI was also checked, as RFI may be another reason for retrieval not being attempted. The percentage of brightness temperature measurements in L1c products for any given node where the presence of RFI was suspected was obtained by summing N_RFI_X and N_RFI_Y and dividing by M_AVA0. The spatial average of this percentage was computed over the Twente and the Maqu region.

Confidence and science flags were analyzed for all the nodes where soil moisture was retrieved, to obtain information about the performance of the retrieval algorithm and the input information provided to the L-MEB model, such as the flag indicating a poor fit between modeled and observed brightness temperature, the flag for snow presence and the flags regarding land cover (*i.e.*, forest flag). This was done by counting the number of nodes where the retrieval was carried out and where a flag of interest was raised. Then, the obtained number was divided by the total number of nodes with data available and the result was expressed as a percentage. The presence of snow, as well as of forest, might be related to retrieval inaccuracies, due to the difficulties in modeling their contribution to the brightness temperature.

Another important factor that might have affected soil moisture retrieval is the accuracy of the land cover map, as, on the base of this information, the dominant land cover type at any given node was determined and the appropriate parameterization of the L-MEB model selected (as mentioned in Section 2.3). For this reason, the two maps used to obtain the ECOCLIMAP cover types, the UMd and the IGBP-DIS map, were compared to the MICLCover map over the Maqu region. Unfortunately, no detailed land cover map is currently available for the Twente region. In order to simplify the comparison between the maps, some cover types were grouped into generic classes, such as forest, shrubland and cropland.

Finally, the determination coefficient, *R^2^*, and the root mean square error, *rmse*, between the SMOS soil moisture spatial average and the *in situ* spatial average were computed considering the complete time series of SMOS data, as well as considering only the data covering at least half the area of interest. Moreover, the same computation was carried out disregarding the SMOS nodes covering some targets that can negatively affect the retrieval, such as, mountainous areas and water bodies, in the Maqu region, and forested areas and urbanized areas, in the Twente region. *R^2^* and *rmse* were also computed only for the period of the year when no snow and frozen soils were present (May–September for Maqu and March–December for Twente), to investigate if the correlation between satellite and *in situ* data depended on the season.

## 4. Results and Discussions

### 4.1. Time Series Comparison

**Figure 3.**
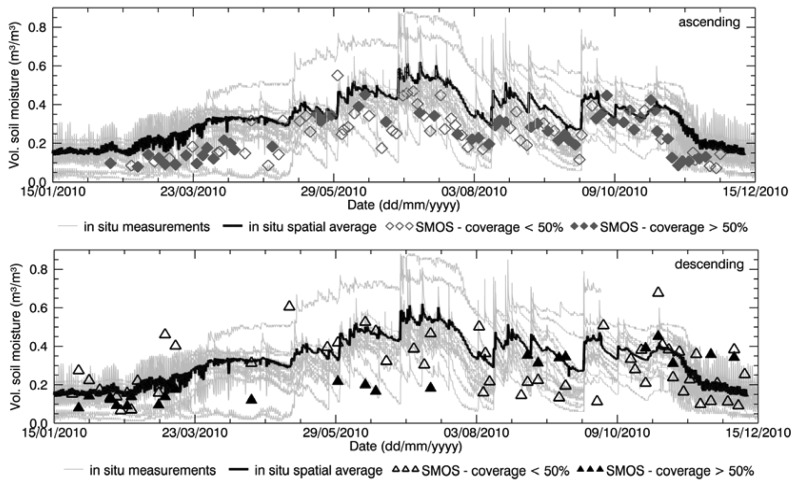
Spatial average of SMOS soil moisture obtained from ascending overpasses (**top**) and from descending overpasses (**bottom**) over the Maqu region compared with *in situ* average soil moisture and individual measurements. Empty symbols indicate that no SMOS products were generated over more than half the region, whereas filled symbols refer to good coverage.

**Figure 4.**
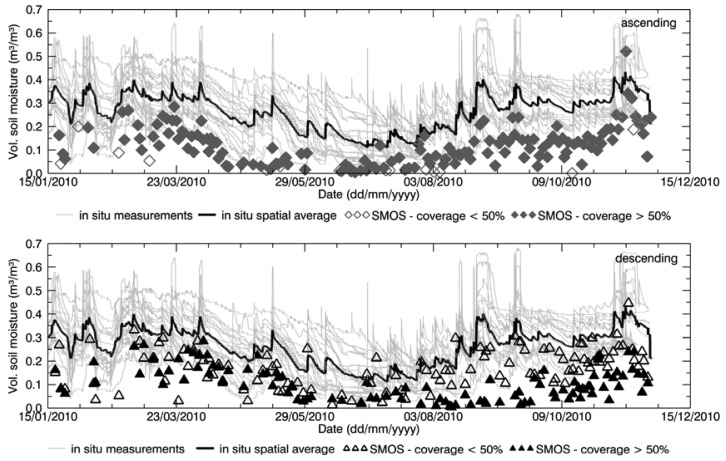
Same as Figure 3 but for the Twente region.

The time series of SMOS data spatially averaged over the area of interest and the time series of the *in situ* average were compared, as shown in Figure 3 for the Maqu region and in Figure 4 for the Twente region. The comparison was done separately for ascending and descending products.

The seasonal behavior of SMOS L2 products is generally in agreement with the soil moisture behavior for both the Maqu and the Twente region. SMOS data on average show relatively low values in the Maqu region in winter with an increase in the monsoon season, following the *in situ* behavior. Instead, in the Twente region the *in situ* data show higher soil moisture values in winter and a decrease in summer with the SMOS data depicting a similar pattern.

However, noise was found in the satellite-derived products, consisting of variations in the SMOS estimates not corresponding to *in situ* data variations, and affecting the agreement between the two datasets. In both regions, the descending products are noisier than the ascending ones. Contribution to the noise is certainly partly due to the fact that several SMOS products covered only partially the area of interest, where the soil moisture retrieval in the rest of the area was unsuccessful. This might affect the validation results, as the SMOS data covering part of the area were compared with the spatial average of the complete network. The empty symbols in Figure 3 and 4 indicate when SMOS products were not generated for at least half of the area, whereas filled symbols refer to a good coverage. The figures show that several SMOS products only partially covered the Maqu region, as well as the Twente region. In general, the two figures show that retrieval was more problematic in the Maqu region than in the Twente region, as data are missing more frequently over the Chinese site with more gaps in the time series, than over the Dutch site. The reason for data in space and time having been missed is investigated in Section 4.2. However, the agreement between the variations found in SMOS data and *in situ* data does not improve by leaving out the products with partial coverage of the area of interest.

From the time series comparison one can also conclude that the SMOS soil moisture is affected by general underestimation, which appears to be larger for the ascending products over both Maqu and Twente, than for the descending ones. Though with a bias, the SMOS data fall within the variability range of the *in situ* data for the Maqu region. However, for the Twente region several SMOS data are outside the *in situ* variability range.

The SMOS mission commissioning phase ended in May 2010, therefore it might be that the data of the first few months of the year have a low quality. However, the agreement between satellite-derived and *in situ* data does not show any clear dependency on this factor. The underestimation, as well as the noise, does not occur in a specific period of the year or for a particular soil moisture variability range.

The reasons for the noise and underestimation in the SMOS soil moisture products, as well as for the data missing in space and time, are investigated in the following sections. Confidence and science flags were analyzed to obtain information about retrieval algorithm characteristics and problems occurring in the retrieval process.

### 4.2. Unsuccessful Retrieval Case

The reason of an unsuccessful retrieval over part of or the whole area of interest was investigated by checking the flags which indicate the supposed presence of frozen soil and RFI and checking the flags which indicate the performance of the retrieval algorithm, such as the flag for out-of-range retrieved values and the flag for high retrieval error. Therefore, in this section only the SMOS nodes without retrieval are included in the analysis.

**Figure 5.**
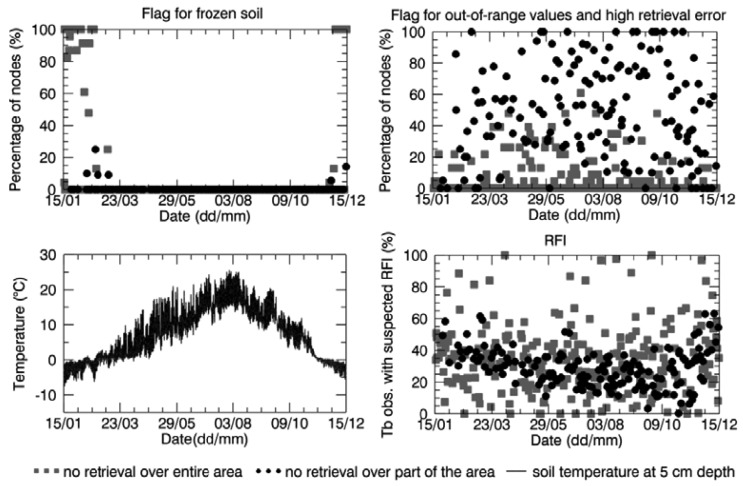
Percentage of SMOS grid nodes in the Maqu region, where the flag for frozen soil (**top-left**) and for out-of-range values and high retrieval errors (**top-right**) were raised. Spatial average of soil temperature measured in the Maqu region at 5 cm depth (**bottom-left**). Percentage of disregarded SMOS brightness temperature observations because of suspected RFI, averaged over the Maqu region (**bottom-right**), when there was no retrieval. Legend refers to all figures.

**Figure 6.**
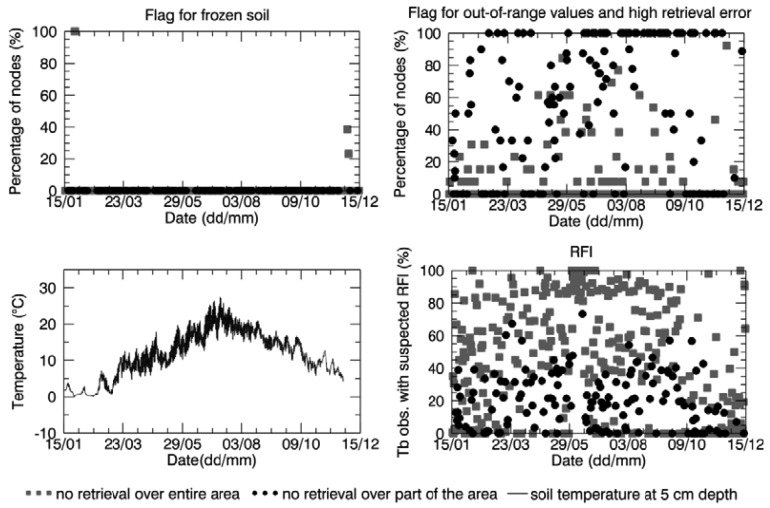
Same as Figure 5 but for the Twente region.

As mentioned in Section 2.3, no retrieval is performed when the soil is frozen. Figure 5 (top-left) shows the percentage of nodes included in the Maqu region with a raised frost flag over the total number of nodes in the region lacking retrieval, for both the case of no retrieval over a part of the area and the case of no retrieval over the whole area. The same plot is presented for the Twente region in Figure 6. Figure 5 shows that the complete area of the Maqu network was characterized by the frost flag several times in winter, resulting in no data, whereas the Twente region was not considered to be covered by frozen soil except for a few days in January and December. When the soil moisture was only retrieved over part of the area, frozen soil was not the main cause of unsuccessful retrieval for the rest of the area.

However, the analysis revealed that for several products of the winter season when no soil moisture was retrieved, the Maqu region was completely frost flag free or only partially covered. The absence of the flag for frozen soils in winter over the whole or part of the Maqu area is in disagreement with the *in situ* measurements. The spatial average of the soil temperature measured *in situ* at 5 cm depth in the Maqu region is shown at the bottom left of Figure 5. The measurements at the Maqu site show that the soil temperature at 5 cm depth dropped below zero from January to February and from November to December 2010 at all sites of the network, meaning the upper layer of the soil was homogenously frozen in winter, whereas in March and October the frost conditions might not be spatially homogenous, though still present. The soil froze several times in January and February in the Twente region as well, as shown in Figure 6, but the frost flag was raised only once in the SMOS data. As mentioned in Section 2.3, the frozen soil flag is raised when the surface temperature falls below a certain threshold, input that is provided by the ECMWF model. Therefore, it is possible that surface temperatures provided to the SMOS L2 processor were not accurate, but further analysis would be needed to investigate this issue.

The main reason for unsuccessful retrieval, in particular when there is a partial SMOS data coverage over the area of interest, was found to be related to retrieval algorithm performance. Indeed, for most of the nodes where no data were retrieved, the confidence flags indicated that the retrieval attempt was unsuccessful because the retrieved parameters were out of range or the error of retrieval exceeded the maximum threshold (see Figures 5 and 6). However, it was not possible to find further information in the L2 products as to what exactly caused this and at which level of the retrieval processing the problem occurred.

The suspected presence of RFI might be another reason for disregarding some SMOS data in the retrieval process, therefore the SMOS L2 product bands related to RFI were analyzed. The number of disregarded SMOS L1c data per node because of suspected RFI, averaged over the Maqu and Twente region, and expressed as a percentage of the total number of brightness temperature observations (obtained as described in Section 3), is plotted in Figure 5 for Maqu and Figure 6 for Twente. The presence of RFI is definitely much higher in the Twente region than in the Maqu region. At the Dutch site it occurred several times that almost all the SMOS observations were affected by RFI, in particular when no retrieval was attempted over the entire network area. This was not the case for the Maqu region, where the site was suspected to be completely covered by RFI for only a few days. However, the Maqu region was not free of RFI on other days. Indeed, an average of 30% of SMOS observations over the nodes without retrieval was considered to be affected by this interference.

### 4.3. Analysis of the Retrieved Value Inaccuracy

The SMOS soil moisture data were further analyzed, in order to investigate the causes of noise and inaccuracy. Therefore, different flags and bands of the SMOS products were checked for all nodes where soil moisture was retrieved, allowing understanding of which input information was used in the retrieval process that might have caused the noise.

**Figure 7.**
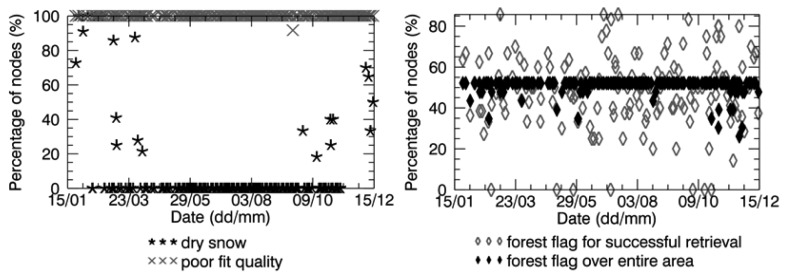
Percentage of SMOS grid nodes (over the total number of nodes where soil moisture was retrieved) in the Maqu region, where the flag of dry snow, poor fit quality and forest were raised.

**Figure 8.**
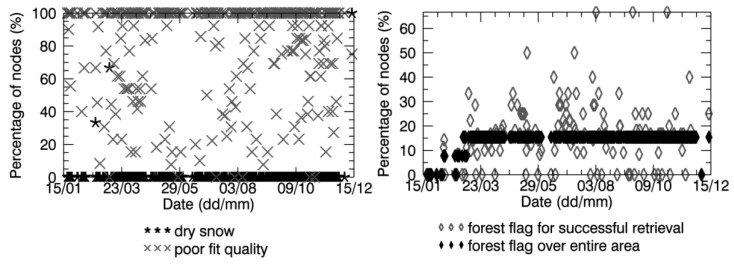
Same as Figure 7 but for the Twente region.

Figures 7 and 8 show that in all the data retrieved over the Maqu region and in most of the data retrieved over the Twente region the flag indicating a poor fit between modeled brightness temperature and SMOS observations was raised. This revealed that the performance of the retrieval algorithm were not optimal over either sites, probably due to inaccurate inputs.

As already mentioned in Section 4.2, the surface temperature used as input to the retrieval algorithm was very probably not accurate, in particular in winter, implying an inaccurate detection of frozen soils. According to the *in situ* measurements (Figures 5 and 6), the soil surface was homogenously frozen in January and February and in November and December in the Maqu region, and in February for several days in the Twente region. Therefore, no SMOS soil moisture data were expected in these periods. However, several data are available, as the frozen soil was not correctly detected. Dry snow was sometimes assumed to be present (Figures 7 and 8), but retrieval was performed as in the nominal conditions, as the snow was considered transparent (Section 2.3).

Frozen soils and snow might have caused an inaccurate retrieval not only in the mentioned periods, but also in the periods shortly before and shortly after, as the region of interest could have been partially frozen or partially covered by snow.

#### 4.3.1. Land Cover Information

Another important input to the SMOS retrieval algorithm is land cover. For this reason, the flags indicating the land cover assumed to be present in each node of the SMOS products were analyzed. Figures 7 and 8 plot the percentage of nodes where the forest science flag was raised over the total number of SMOS nodes in the Maqu region and in the Twente region, respectively, as well as the percentage of nodes with a raised forest flag where the retrieval was carried out successfully. The figures show that half of the nodes in the Maqu region and approximately 15% of the nodes in the Twente region were considered to be covered by forest in most of the SMOS data and that this number varied with time. The assumption of forest cover in 15% of the nodes in the Twente region is realistic, as is possible to see in the color composite image of Figure 2, whereas the land cover assumed for the Maqu region is not correct.

Therefore, there are two important issues that arise with the analysis of the forest flag. The first issue concerns its variability in time. If the number of nodes with a forest flag set to one is variable in time, it means that a forest fraction is sometimes assumed to be present in a certain node and other times not. The reason of the variability in time of the forest flag for a specific node can be found in the method used in the SMOS retrieval algorithm to determine the dominant land cover at each node, as is explained in Section 2.3. The consequence is that when the forest flag is raised at a certain node, the emissivity simulated by the L-MEB model includes a forest contribution, and when the forest flag is not raised, only low vegetation contribution is included. This can certainly affect the consistency in time of the soil moisture retrieval at a specific node.

**Figure 9.**
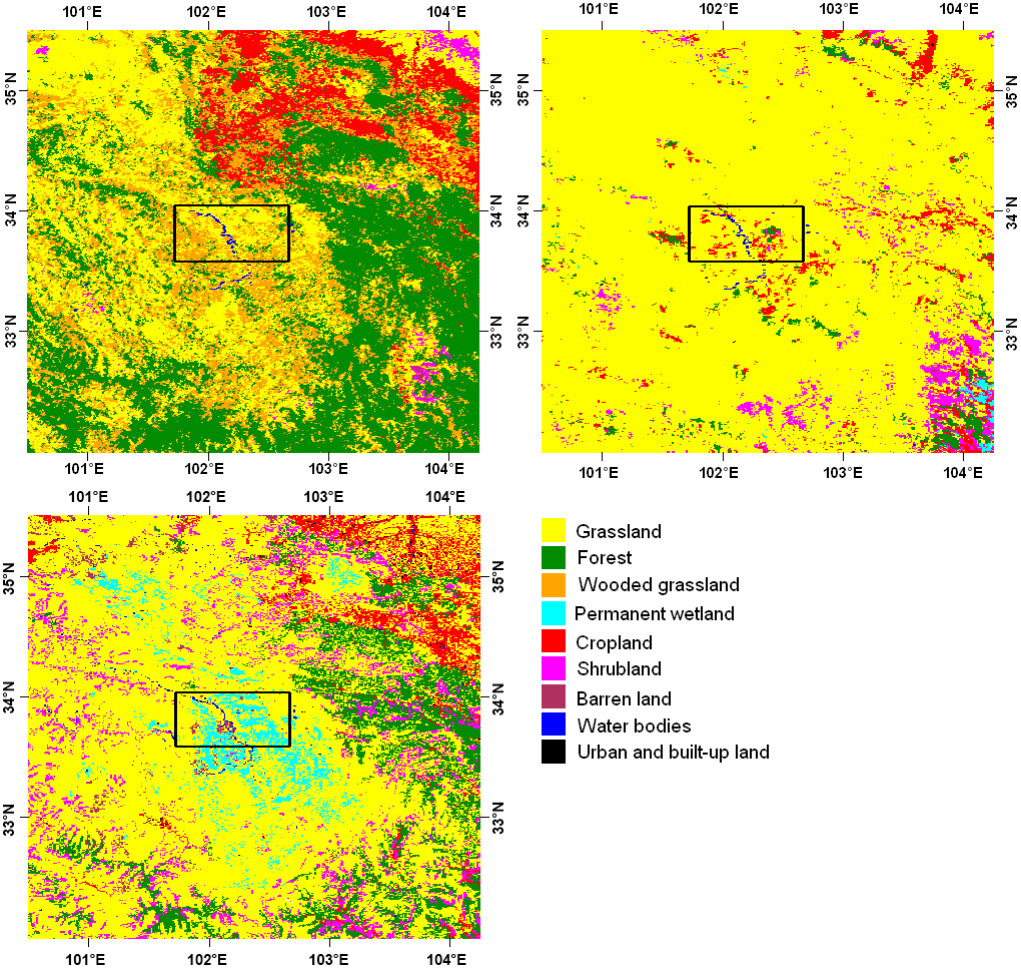
UMd map (**top-left**), IGBP-DIS map (**top-right**) and MICLCover map (**bottom-right**) over the Maqu region and surrounding areas. The black rectangle highlights the Maqu network location.

The second issue concerns the incorrectly classified land cover in the Maqu region. According to information collected *in situ*, homogenous grasslands cover the network area and its surrounding areas within tens of kilometers, and the valley near to the Yellow River is characterized by wetlands. However, the forest flag is raised for half of the nodes in this region and the wetland flag is never raised. For this reason, accuracy of the land cover information used by the SMOS L2 processor was investigated. The two cover maps on which the ECOCLIMAP is based, the UMd map and the IGBP-DIS map, were compared with an independent land cover map, the MICLCover map. Figure 9 shows the three maps for the Maqu region.

According to the UMd map the Maqu region is mainly covered by grassland and wooded grassland, with some small areas of forest, and according to the IGBP-DIS map there are no wetlands. Both maps give inaccurate information, whereas more realistic information can be obtained from the MICLCover map, which shows the presence of mainly grasslands and wetlands, with small areas of barren and low vegetated lands. However, despite the UMd map's inaccuracy, if only the network area had been considered, the dominant vegetation type obtained from it for all nodes would probably be grassland/wooded grassland without any forest. Instead, the SMOS L2 processor working area for determination of the dominant land cover is 123 km × 123 km large, thus the surrounding area within at least one degree around the network has to be taken into account in the present discussion. The UMd map shows the presence of dense and large forests at less than 20 min of a degree east of the network area, as well as of forest mixed with grassland and wooded grassland to the south and west. The forested areas shown in the UMd map contributed to the determination of the dominant vegetation type when the SMOS view covered them. When the presence of forest was detected, then the forest flag was raised. The nodes with raised forest flags are mainly located near both the eastern and the western borders, which means that not only the dense forest in the eastern part of the UMd map affected the dominant land cover type determination in the network area, but the forest in the western part did as well. The nodes located in the centre of the Maqu region were not characterized by a raised forest flag. The percentage of land covered by forest in the areas surrounding the network is much lower on the MICLCover map, which corresponds with the information collected *in situ*. If the MICLCover map had been used in the SMOS L2 processor, the number of nodes with a forest flag would probably be much reduced, although the flag would be still raised in a few eastern nodes due to the presence of the forest to the east of the Maqu network.

#### 4.3.2. Radio Frequency Interference

The quality of the L1c SMOS products, used as input to the L2 processor, strongly affects the accuracy of the retrieved soil moisture. As mentioned in the introduction, RFI is currently the most important cause of decreased quality of both the SMOS L1c and L2 products.

**Figure 10.**
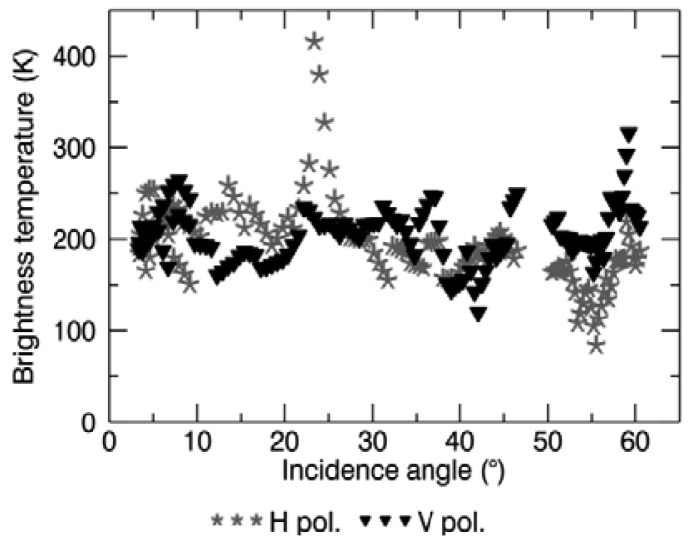
Angular pattern of the brightness temperature of one node in the Maqu region on 5 July 2010.

A preliminary and not comprehensive analysis of the SMOS L1c brightness temperature data was carried out to investigate the possible presence of RFI over the Maqu and the Twente region. A proper rotation was applied to the data, converting the brightness temperature in the antenna reference (X and Y polarization) to that in the Earth reference (H and V polarization). The analysis revealed that for both sites several data are characterized by out of range brightness temperature values, indicating the presence of RFI in the area. Those data were generally filtered out and disregarded by the retrieval process. However, it was also found that several data are characterized by brightness temperatures with acceptable values, but with an unexpected feature in the angular pattern. The angular pattern of both horizontal and vertical polarized brightness temperature was found to have abrupt variations corresponding to small incidence angle increases, instead of the expected smooth variations. All the data collected over the Maqu region, as well as all the descending plus several ascending data over the Twente region, are characterized by this feature. Figure 10 shows an example of the irregular angular pattern of the horizontal and vertical polarized brightness temperature measured at one of the nodes in the Maqu region on July 5, 2010. As explained in [21,22], the brightness temperature angular pattern can be strongly affected by the presence of RFI. Moreover, if the RFI is weak, the observed brightness temperature might range between acceptable values, while the angular pattern is irregular. In this situation, the filtering of RFI-affected observations is not straightforward and studies about RFI detection are ongoing. The use of L1c data improperly filtered might be the cause of less accurate soil moisture retrieval.

**Figure 11.**
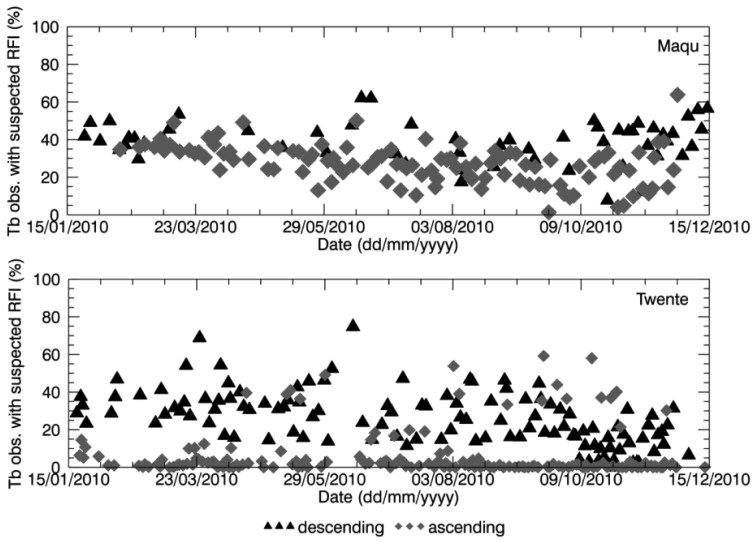
Disregarded SMOS brightness temperature observations because of suspected RFI, averaged over the Maqu (**top**) and the Twente (**bottom**) region and expressed as a percentage of the total number of SMOS observations.

Several SMOS observations were disregarded in the soil moisture retrieval process. However, in particular for those dates when a large number of SMOS data was suspected to have been affected by RFI, one cannot exclude that RFI might also have been present in the remaining data used for the retrieval. As the RFI caused a bias in the measured brightness temperature, consequently the retrieved soil moisture might be affected by a bias as well. Most of the time, when the brightness temperature bias due to RFI is positive, then the corresponding bias in the soil moisture is negative. This could be one of the reasons for the dry bias observed in the SMOS soil moisture products. Figure 11 shows the number of disregarded SMOS L1c data per node because of suspected RFI, averaged over the Maqu and the Twente region, and expressed as a percentage over the total number of brightness temperature observations (obtained as described in Section 3). Only the nodes where soil moisture was retrieved were considered for this plot.

Over the Maqu region, approximately 40% of the SMOS descending observations were disregarded in the retrieval process, as suspected of being affected by RFI. The number of ascending data not used in the retrieval was approximately 40% at the beginning of 2010, but slowly decreased during the year. Over the Twente region, the descending data were clearly more affected by RFI than the ascending data. The average percentage of disregarded descending observations was approximately 30%, but could on occasion rise above 60%. Whereas, most of the ascending passes were free of RFI in the Twente region in 2010.

### 4.4. Time Series Correlation

**Figure 12.**
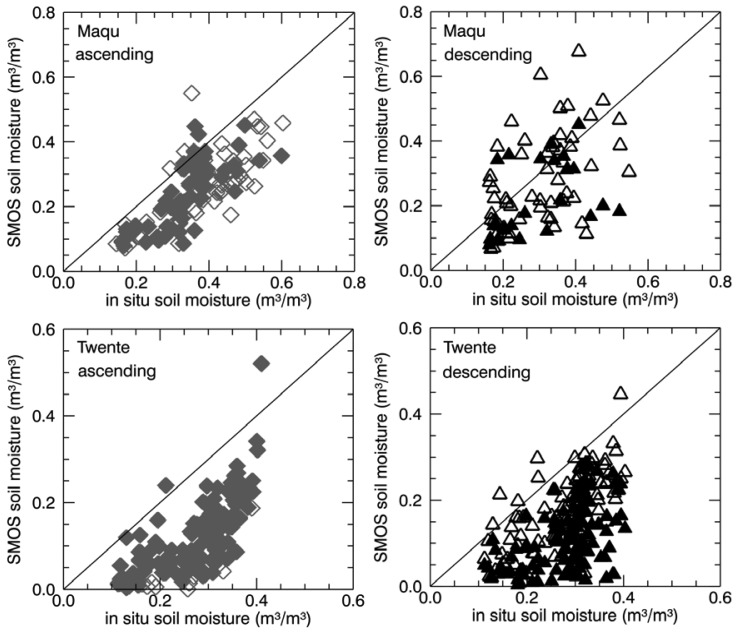
SMOS soil moisture averaged over the Maqu (**top**) and the Twente (**bottom**) region as a function of *in situ* average soil moisture at the satellite acquisition time, for ascending passes (**left**) and for descending passes (**right**). Empty symbols indicate that for more than half of the area no SMOS products were generated, whereas filled symbols refer to a good coverage.

In order to have a clearer view of the match between retrieved and measured soil moisture, the SMOS soil moisture spatial average was compared with the *in situ* measurements collected at the time of the satellite acquisitions, as shown in the scatter plots in Figure 12. Moreover, Table 1 reports the determination coefficient, *R^2^*, and the root mean square error, *rmse*, between the two time series. The analysis was done separately for ascending and descending data.

For both the Maqu and the Twente region, there is closer agreement of the SMOS soil moisture retrieved from ascending data and the *in situ* data, than from descending data, though *R^2^* remains relatively low, *i.e.*, 0.55 for the Maqu region and 0.51 for the Twente region. The plots show that the dry bias in the SMOS retrieval is more evident in the ascending case than in the descending case, but that the data are more spread out in the latter case than in the former. A quantification of the dry bias of the SMOS data was obtained with the computation of the *rmse*. Products retrieved from ascending data over the Twente site show on average a dry bias of approximately 0.17 m^3^/m^3^, whereas those over the Maqu site show a bias of approximately 0.13 m^3^/m^3^. The correlation and the dry bias only slightly improves, if the SMOS data covering less than half the area of interest are disregarded (empty symbols in Figure 12), though not for the descending data over Maqu. Disregarding the periods of possible presence of frozen soils and snow does not improve the match between the two datasets over Maqu and only slightly increase the determination coefficient over Twente. Moreover, in order to check if the presence of mountainous areas, the river and the cities as well as the presence (or assumed presence) of forest in the observed area affected the agreement between satellite-derived and *in situ* data, the *R^2^* and *rmse* were also computed, disregarding the nodes covering these targets in the SMOS spatial average. Table 1 shows that the *R^2^* and *rmse* does not significantly change when the mountainous areas and the river of the Maqu region and the largest cities of the Twente region are excluded by the computation. Moreover, *R^2^* and *rmse* does not improve when the nodes with a raised forest flag are excluded from the SMOS spatial average. This leads to the conclusion that noise and bias of the SMOS soil moisture data over Maqu and Twente region are not due to the presence of these targets and are not related to the inclusion in the L-MEB model of a forest contribution. Moreover, they are not limited to the period of frost and snow.

The difference between the depth of the soil layer contributing to the brightness temperature measured by SMOS radiometer and the soil depth where the measurements were carried out certainly affects the relationship between satellite-derived and *in situ* data. Measuring soil moisture at deeper layers than the penetrating depth of the microwave radiation should lead to an underestimation in the dry season (as the upper soil layers are dryer than the deeper layers) and an overestimation in the rainy season or soon after a rainy event (as the upper layers are wetter than the deeper ones), but this is not the case for the SMOS data, as the dry bias is quite systematic in all seasons. Therefore, this effect does not explain the SMOS bias.

The *rmse* found over the Maqu and Twente region are much higher than the SMOS mission requirements for soil moisture observations, *i.e.*, 0.04 m^3^/m^3^ [2]. The dry bias and noise in the SMOS data are not specific for the Maqu and the Twente sites, as they were also found at the upper Danube catchment (southern Germany) [23], at two sites in Italy and one in Luxemburg [24], at the REMEDHUS network area in Spain [25] and at several sites of the SCAN network in the U.S. [26]. However, there are also sites where bias was almost completely absent, as it was shown at the Walnut Gulch (AZ) watershed, the Little Washita (OK) watershed and the Little River (GA) watershed in the U.S. [27] as well as at some of the sites of the SCAN network [26]. Considering all these studies one could conclude that the dry bias is not dependent on a specific land cover, surface condition or season, though it might be related to the presence of RFI. However further analyses are needed to understand the cause.

## 5. Conclusions

The validation of SMOS L2 soil moisture products is a crucial step before the planned further refinement of the retrieval algorithm and data reprocessing. Several validation campaigns are expected to contribute to the understanding whether inaccuracies are present in the retrieved values and where the retrieval limitations lie. This study intended to contribute to the validation of the currently available SMOS L2 products, by comparing them with *in situ* data collected at the Maqu region (China) and the Twente region (The Netherlands) in 2010.

The seasonal behavior of the SMOS products is generally in agreement with the *in situ* data over both regions. The validation analysis resulted in an *R^2^* of 0.55 and *rmse* of 0.13 m^3^/m^3^ over the Chinese site and an *R^2^* of 0.51 and *rmse* of 0.17 m^3^/m^3^ over the Dutch site for the ascending pass SMOS products. The agreement between the satellite-derived and *in situ* data is less when the descending pass data are considered, with an *R^2^* of 0.24 and *rmse* of 0.13 m^3^/m^3^ for the Maqu region and an *R^2^* of 0.41 and *rmse* of 0.12 m^3^/m^3^ for the Twente region. The relatively low *R^2^* and high *rmse* are due to the presence of noise and a systematic dry bias in the SMOS data.

It was found that the retrieval is more problematic in the Maqu region than in the Twente region. Several SMOS L2 data over the Maqu region are missing or cover only a small fraction of the network area, as the retrieval attempt was unsuccessful in the remaining part. However, the *R^2^* does not improve when the data covering less than half of the area are disregarded. It was also found that the presence of mountainous areas, water bodies, forest and cities does not affect the retrieval accuracy over these regions, as the *R^2^* and *rmse* do not improve when the SMOS nodes covering these targets are disregarded.

The presence of frozen soils and snow in the Maqu region, as well as in the Twente region, might be one of the causes of low retrieval accuracy in winter, amplified by the fact that the input information about frozen surfaces (*i.e.*, surface temperature) used in the SMOS retrieval process was probably inaccurate. Further analyses of the surface temperature provided by the ECMWF model would be needed to assess its accuracy, as well as the impact of this factor on the soil moisture retrieval, not only in winter but also in the other seasons.

Another important input which resulted in inaccuracies for the Maqu region (but not for the Twente region) is the land cover type. The wrong land cover type being used in the SMOS retrieval over the Maqu region is due to both the inaccurate land cover information provided by ECOCLIMAP and the contribution of the land cover of the neighboring areas in the determination of the dominant land cover type for the nodes within the network area. A dedicated study assessing the SMOS retrieval sensitivity to land cover map accuracy, by comparing soil moisture retrieved using different land cover maps (such as MODIS, GLC2000 and GLOBCOVER land cover maps), would be needed. Though improving the accuracy of the input land cover map might improve the accuracy of the SMOS products over the Maqu region, the contribution of neighboring areas, to both the determination of the land cover in a specific node and the brightness temperature measured for that node, cannot be avoided when the satellite sensors have a low resolution, as the SMOS satellite has.

The presence of RFI was detected over both regions. This is probably the most important factor causing noise and bias in the SMOS soil moisture products. Several L1c data were filtered out of the retrieval process. However, it was found that an irregular angular pattern in the brightness temperature characterizes many of the L1c data employed in the retrieval. This is most probably due to a weak and not well filtered RFI. The SMOS ascending data over the Twente region are less affected by RFI than the other dataset, however they do not particularly show better agreement with the *in situ* data than the other dataset does. The SMOS soil moisture products will certainly benefit from the use of an improved RFI detection and filter method, as is currently under investigation.

Both further L1 data re-processing and further refinement of the soil moisture retrieval algorithm on the base of the validation campaign results are planned. Therefore, it is expected that the SMOS soil moisture data quality will rapidly be improved.

## Figures and Tables

**Table 1.** Determination coefficient (*R^2^*) and root mean square error (*rmse*) between SMOS ascending and descending soil moisture data and *in situ* measurements, considering all the data available, the data that cover at least half the area of interest, the data covering flat areas only, all the data except those covering the water bodies, the forest and the urban areas or collected in the winter/frost period.

	**Ascending**		**Descending**	

**MAQU**	**R^2^**	**rmse (m^3^/m^3^)**	**R^2^**	**rmse (m^3^/m^3^)**
All data	0.55	0.13	0.24	0.13
Data with coverage > 50%	0.57	0.12	0.12	0.09
Data over flat areas	0.50	0.14	0.20	0.14
Data without water bodies	0.55	0.14	0.27	0.13
Data May–September	0.33	0.15	0.03	0.17
Data without forest flag	0.48	0.14	0.22	0.14

**TWENTE**				

All data	0.51	0.17	0.41	0.12
Data with coverage > 50%	0.52	0.16	0.42	0.12
Data without forest flag	0.52	0.17	0.40	0.12
Data without cities	0.52	0.16	0.43	0.12
Data March–December	0.53	0.16	0.48	0.11
